# Comparative performance of IP-10-Based assays for immune detection of *Mycobacterium tuberculosis* infection: a cross-sectional evaluation

**DOI:** 10.3389/fmed.2026.1886171

**Published:** 2026-08-17

**Authors:** Alexandru Stoichita, Madalina Burecu, Camelia Nita, Cristina Teleaga, Alexandru Daniel Radu, Mihaela Mihai, Beatrice Mahler, Elmira Ibraim

**Affiliations:** 1 Clinical Research Department, Marius Nasta Institute of Pneumophthisiology, Bucharest, Romania; 2 Department of Pulmonology, Carol Davila University of Medicine and Pharmacy, Bucharest, Romania; 3 TB Dispensary, Marius Nasta Institute of Pneumophthisiology, Bucharest, Romania; 4 Faculty of Economic Cybernetics, Statistics and Informatics, Bucharest University of Economic Studies, Bucharest, Romania; 5 Clinical Department 1, Marius Nasta Institute of Pneumophthisiology, Bucharest, Romania

**Keywords:** assay agreement, IP-10, latent TB infection, QuantiFERON-TB Gold plus, RIDA^®^QUICK TB, RIDA^®^SCREEN TB, tuberculosis infection

## Abstract

**Background:**

Reliable immune-based detection of *Mycobacterium tuberculosis* infection remains challenging, particularly in TB contacts and individuals with immune dysregulation. IP-10 is produced at substantially higher concentrations than IFN-γ and represents a promising biomarker for enhancing IGRA-based immune detection. This study evaluated the comparative performance of two IP-10-based assays, RIDA^®^QUICK TB and RIDA^®^SCREEN TB, in relation to QuantiFERON-TB Gold Plus.

**Methods:**

This cross-sectional comparative evaluation enrolled 99 adults: 49 with culture-confirmed active pulmonary TB, 30 close TB contacts, and 20 individuals with autoimmune disease. All participants underwent RIDA^®^QUICK TB, RIDA^®^SCREEN TB, and QFT-Plus testing. Because no independent gold standard exists for latent tuberculosis infection, assay performance was assessed using positivity rates, overall percent agreement, positive percent agreement, negative percent agreement, Cohen’s kappa, McNemar testing, and discordance analysis. In the culture-confirmed active TB subgroup, the proportion of positive immune-test results was reported descriptively.

**Results:**

Among culture-confirmed active TB cases, RIDA^®^QUICK TB was positive in 42/49 participants, while RIDA^®^SCREEN TB was positive in 45/49 participants, compared with 35/49 for QFT-Plus. This estimate reflects immune-test positivity among culture-confirmed active TB cases and should not be interpreted as sensitivity for latent tuberculosis infection. Across the full cohort, both IP-10-based assays yielded higher positivity rates than QFT-Plus, particularly in the active TB subgroup. Agreement with QFT-Plus varied by clinical subgroup and was strongest among TB contacts, supporting the immunological concordance of the evaluated platforms in this population. ROC analyses using QFT-Plus as the comparator demonstrated good immunological concordance between the IP-10-based assays and QFT-Plus-defined immune status. These analyses evaluate agreement between immune-response platforms and do not represent diagnostic accuracy against a definitive microbiological standard.

**Conclusion:**

RIDA^®^QUICK TB and RIDA^®^SCREEN TB demonstrated encouraging performance, with high positivity among culture-confirmed active TB cases and clinically relevant agreement with QFT-Plus across distinct participant groups. These findings support the potential role of IP-10-based assays as complementary immune-based tools for TB infection evaluation, particularly in settings where enhanced immune-response detection may be useful. Larger, clinically characterized and longitudinal studies are warranted to further define specificity, predictive value, and integration into LTBI screening strategies.

## 1 Background

Respiratory pathogens are a significant contributor to illness and death, both in the European Union and worldwide, and will persistently pose a risk to public health during future outbreaks and global health crises (1). The COVID-19 pandemic underscored the pivotal role of diagnostics in monitoring and testing individuals irrespective of symptomatology, enabling timely public health interventions to curb viral transmission. Early identification of infection is essential to reduce both morbidity and mortality. Given the overlapping clinical presentation of respiratory infections, the use of rapid diagnostic methods is critical for accurate pathogen identification, guiding appropriate treatment strategies and containment measures. This is particularly relevant as respiratory infection syndromes account for a substantial proportion of deaths attributable to antimicrobial resistance worldwide (2).

Tuberculosis (TB), caused by *Mycobacterium tuberculosis* (Mtb), remains one of the world’s most lethal infectious diseases despite available preventive and curative therapies. In 2024, TB remained the leading cause of death from a single infectious agent worldwide. Globally, an estimated 10.7 million people developed TB, and approximately 1.23 million deaths were attributed to the disease, including around 150,000 deaths among people living with HIV, reflecting a modest decline compared with 2023 but a burden that remains unacceptably high (3).

In the WHO European Region, sustained progress has been observed over the past decade. According to the ECDC/WHO Tuberculosis Surveillance and Monitoring in Europe 2025 report, EU/EEA countries reported 38,993 TB cases in 2023, corresponding to a notification rate of 8.6 cases per 100,000 population, while TB-related mortality remained low at the regional level. Compared with 2015, the WHO European Region has achieved an estimated 39% reduction in TB incidence and a 49% reduction in TB deaths, although substantial heterogeneity persists between countries (3, 4).

Romania continues to report the highest TB burden among European Union (EU) member states, despite a sustained decline in incidence and mortality over the past decade. According to the WHO Global Tuberculosis Report 2025, Romania is classified as a lower-moderate TB incidence country, yet its TB notification rates remain substantially higher than the EU/EEA average, which is low and relatively stable. This pattern is corroborated by the ECDC/WHO Tuberculosis Surveillance and Monitoring in Europe 2025 Report, which documents an EU/EEA notification rate of 8.6 cases per 100,000 population in 2023, while Romania continues to contribute a disproportionate share of notified cases, including the highest notification rates among young children, indicative of ongoing transmission. Clinically, TB-related mortality in Romania remains several-fold higher than the EU average, suggesting delayed diagnosis, advanced disease at presentation, and the persistent influence of social vulnerability and comorbidities. Although Romania follows the overall downward epidemiological trend observed in the WHO European Region, these indicators highlight persistent clinical and epidemiological disparities within the EU, underscoring the need for strengthened early case detection, targeted interventions in high-risk populations, and improved continuity of care to further reduce TB burden and mortality (3, 4).

The WHO End TB Strategy aims to eliminate TB by reducing incidence, mortality, and associated suffering, with a major pillar focused on expanding access to preventive treatment for high-risk individuals. Latent TB infection (LTBI) represents a sustained immune response to Mtb antigens in the absence of clinical disease. It is estimated that 25% of the global population is infected. Accurate detection of LTBI is essential to identify individuals who may benefit from TB preventive therapy and thereby interrupt progression to active disease (5).

Currently, there are two classes of tests available for LTBI: interferon-gamma release assays (IGRAs) and the tuberculin skin test (TST). These tests are indirect and rely on a strong immune response to accurately identify individuals who are infected with TB (6).

The TST, although inexpensive and widely available, suffers from limited specificity due to BCG vaccination, environmental non-tuberculous mycobacteria (NTM), and reduced sensitivity in immunocompromised individuals (6).

IGRAs, including QuantiFERON-TB Gold Plus (QFT-Plus) and T-SPOT.TB, offer greater specificity by measuring IFN-γ responses to Mtb–specific antigens and are unaffected by BCG or most NTM. However, IGRAs remain more costly, require stricter pre-analytical handling, cannot distinguish infection from active disease, and may yield false-negative or indeterminate results in early TB, low-burden infection, or immune-dysregulated hosts (7, 8).

IP-10 is a highly promising complementary biomarker for LTBI diagnosis. Although induced downstream of IFN-γ, it is produced in substantially higher concentrations by antigen-presenting cells and can also be stimulated by IL-2, type I IFs, IL-27, IL-17, IL-23, TNF-α and IL-1β. Its markedly higher dynamic range and stable signal-to-background ratio allow more robust detection using ELISA or lateral-flow platforms compared with IFN-γ, supporting IP-10 as a reliable indicator of antigen-specific cell-mediated immune activation (9–15).

The utilization of assays that evaluate IP-10 could have the potential to enhance LTBI detection. In the current study we aimed to use such assays using the RIDA^®^QUICK TB and the RIDA^®^SCREEN TB tests.

Currently, there are limited published data evaluating the comparative performance of R-Biopharm AG-developed IP-10-based assays in relation to established IFN-γ-based IGRAs such as QuantiFERON-TB Gold Plus. Because no definitive gold standard exists for latent tuberculosis infection, studies comparing immune-based assays should be interpreted primarily as agreement and concordance analyses rather than definitive diagnostic accuracy assessments.

The aim of this study was to generate independent comparative data on the performance of R-Biopharm AG-developed IP-10-based assays in three clinically relevant groups: individuals with culture-confirmed active pulmonary TB, close TB contacts, and individuals with autoimmune disease. Assay performance was assessed against microbiological confirmation in the active TB subgroup and by immunological agreement with QFT-Plus across the full cohort.

## 2 Objectives

### 2.1 Primary objective

To evaluate the comparative performance of two IP-10-based assays, one ELISA-based and one lateral-flow-based, for immune detection of *Mycobacterium tuberculosis* infection, using microbiological confirmation in active TB cases and QFT-Plus as an immunological comparator in non-active TB groups.

### 2.2 Secondary objectives

1. To assess the agreement between the R-Biopharm AG-developed assays and QFT-Plus among close TB contacts.

2. To analyze the concordance between the R-Biopharm AG-developed assays and QFT-Plus in individuals with autoimmune disease.

### 2.3 Study design and setting

This was a cross-sectional comparative assay evaluation conducted in three clinically defined participant groups at a tuberculosis dispensary of the Marius Nasta Institute of Pneumophtiziology in Bucharest, Romania.

## 3 Material and methods

### 3.1 Study population

The study intended to include 100 patients, aged 18 years or older, divided into 3 groups: one group of 50 patients with active pulmonary TB disease, one other of 30 patients with presumptive LTBI (TB contacts) and the third of 20 patients with an autoimmune illness.

Given the absence of robust prior data for IP-10 assays performance, a formal power calculation was not feasible; a target sample of 100 participants was selected for selected for exploratory evaluation of assay positivity, agreement, and concordance across clinically relevant subgroups.

### 3.2 Inclusion criteria

- Adults (aged 18 years or older)

- With active pulmonary TB confirmed by positive Mtb culture from sputum (group 1)

- Close contact of an individual with active pulmonary TB and positive microscopy of sputum (group 2)

- With a documented autoimmune disease diagnostic (group 3)

- Provided consent to participate in the study, after being fully informed.

### 3.3 Exclusion criteria

- Minor (below 18 years old)

- With active pulmonary TB, but negative culture for Mtb from sputum (group 1)

- No documented close contact of an individual with active TB positive for Mtb in sputum microscopy (group 2)

- No documented diagnostic of an autoimmune disease (group 3)

- Treatment for TB disease or LTBI completed within the past 12 months

- Any medication with activity against Mtb taken within 2 weeks before study entry

- Not able or not willing to consent to participate in the study.

The autoimmune disease subgroup included patients with chronic immune-mediated inflammatory disorders, such as rheumatoid arthritis, systemic lupus erythematosus, and other connective tissue diseases. Where available, detailed clinical data were collected regarding disease type, duration, and activity status.

Information on ongoing immunosuppressive therapy was also recorded, including the use of systemic corticosteroids, conventional disease-modifying antirheumatic drugs (DMARDs), and biological agents. Given the known impact of both underlying immune dysregulation and immunosuppressive treatment on T-cell–mediated responses, these variables were considered relevant for the interpretation of immunological assay‘s performance.

### 3.4 Principles of the tests

Whole blood samples were pre-analytically stimulated using RIDA^®^TB Tubes to induce antigen-specific IP-10 release, either by direct venipuncture into stimulation tubes or by transfer from lithium-heparin collected blood, followed by incubation and plasma separation according to the manufacturer’s protocol. For each participant, three plasma conditions (NEG, TEST, POS) were analyzed to ensure assay validity and appropriate immune response control.

The RIDA^®^QUICK TB assay is a rapid immunochromatographic lateral flow assay that detects IP-10 through the formation of an antibody–antigen sandwich complex, visualized via colloidal gold conjugation. Signal intensity is quantified using the RIDA^®^Q3 reader and translated into IP-10 concentrations based on an internal calibration algorithm. The assay has a limit of quantification (LoQ) of 0.25 ng/mL and a limit of detection (LoD) of 0.25 ng/mL.

The RIDA^®^SCREEN TB assay is a quantitative enzyme-linked immunosorbent assay (ELISA) employing monoclonal antibodies directed against IP-10 in a sandwich configuration. Following incubation with biotinylated detection antibodies and streptavidin–peroxidase conjugate, a substrate-driven enzymatic reaction generates a measurable chromogenic signal. Optical density is directly proportional to IP-10 concentration in the analyzed plasma samples. The assay provides semi-quantitative measurements of IP-10 concentrations via the RIDA^®^Q3 reader. The limit of quantification (LoQ) is 0.40 ng/mL, and the limit of detection (LoD) is 0.24 ng/mL.

### 3.5 Reference standards

*Mycobacterium tuberculosis* culture from sputum was defined as the primary microbiological reference standard for the active TB subgroup. GeneXpert and sputum smear microscopy served as secondary bacteriological references.

QFT-Plus, an ELISA-based IFN-γ release assay, was used as an established immunological comparator. However, because QFT-Plus is itself an immune-response assay and does not directly detect viable Mycobacterium tuberculosis, it was not considered an independent diagnostic gold standard. Therefore, comparisons between QFT-Plus and the IP-10-based assays were interpreted as agreement and concordance analyses rather than adjudication of true infection status. The active TB subgroup was used as a culture-confirmed disease-enriched group to explore assay positivity in microbiologically confirmed tuberculosis. Sensitivity estimates derived from this subgroup refer only to immune-test positivity among patients with active culture-confirmed pulmonary TB and are not equivalent to sensitivity for latent tuberculosis infection.

### 3.6 Handling of indeterminate results

Indeterminate results for RIDA^®^QUICK TB, RIDA^®^SCREEN TB and QFT-Plus were reclassified using a composite reference standard of Mtb from sputum: GeneXpert test, smear microscopy and culture. Indeterminate results in confirmed TB cases were coded as positive, reflecting expected immune activation.

Indeterminate results in TB contacts and subjects with autoimmune disease, without bacteriological or radiological evidence of active TB, were coded as negative.

This reclassification strategy was applied solely to minimize analytical exclusions and was not intended to redefine true infection status, acknowledging the potential for incorporation bias inherent to disease-enriched diagnostic studies. The study design did not include a TB-unexposed control group; therefore, specificity and NPV could not be reliably estimated.

### 3.7 Data collection, management and analysis

#### 3.7.1 Data sources

Study data were systematically recorded in a secure electronic database. Source documentation included the following:

- Medical records for demographic and clinical variables
- Registers for TB case notification
- TB laboratory registers for results of microbiological and molecular assays from sputum
- Immunology laboratory registers for tests results

#### 3.7.2 Data analysis

The clinical and laboratory investigators reviewed, analyzed, cleaned the all data into the data source documents and uploaded them into the secure electronic database.

Then, the data have been examined using suitable statistical software, such as Stata and R. For comparisons with the microbiological reference standard in the culture-confirmed active TB subgroup, the proportion of positive assay results was reported as sensitivity. Because this subgroup did not include culture-negative individuals, specificity, NPV, and κ against culture were not interpreted as valid diagnostic accuracy estimates. For assay-to-assay comparisons with QFT-Plus, results were analyzed using overall percent agreement, positive percent agreement, negative percent agreement, Cohen’s κ, McNemar testing, and descriptive discordance analysis. Cohen’s κ values were interpreted using conventional thresholds: ≤0.20, slight agreement; 0.21–0.40, fair agreement; 0.41–0.60, moderate agreement; 0.61–0.80, substantial agreement; and 0.81–1.00, almost perfect agreement (16). These comparator-based metrics were interpreted as immunological agreement rather than definitive diagnostic sensitivity or specificity. Receiver operating characteristic curve analysis was performed as a comparator-based assessment of the ability of the continuous or semi-quantitative IP-10 assay measurements to distinguish QFT-Plus-positive from QFT-Plus-negative immune-response classifications. Because QFT-Plus is itself an indirect host-response assay and does not establish the presence or absence of viable *Mycobacterium tuberculosis*, the resulting AUC values were interpreted as measures of immunological concordance with QFT-Plus-defined status rather than as measures of true diagnostic accuracy. A separate culture-referenced ROC analysis could not be performed in the active-TB subgroup because all 49 participants included in that analysis were culture-positive and no culture-negative reference observations were available. In the absence of both reference-positive and reference-negative cases, specificity, ROC coordinates, and AUC against culture are not estimable. Therefore, the active-TB analysis was limited to the proportion of culture-confirmed cases with a positive immune-test result, reported with 95% confidence intervals. For QFT-Plus interpretation, the 0.20–0.70 IU/mL interval was applied according to the manufacturer’s instructions for use as a predefined low-level/borderline IFN-γ response zone around the standard assay cut-off. This interval was not defined *post hoc* by the investigators and was used only to support standardized interpretation of low-level or discordant QFT-Plus responses. It was not intended to redefine the diagnostic threshold or to serve as an independent reference standard.

## 4 Results

### 4.1 Study population and demographic characteristics

A total of 100 adults were enrolled and allocated into 3 predefined groups: subjects with active pulmonary TB (*n* = 50), close contacts of individuals with microscopy-positive pulmonary TB (*n* = 30) and subjects with an autoimmune disease undergoing evaluation for LTBI (*n* = 20).

One participant in group 1, even positive for GeneXpert test and microscopy from sputum, did not find the inclusion criteria, being that the culture examination did not show growth. Therefore, he was eliminated from the statistical analysis, which has been done on just 49 cases in group 1 and on 99 study subjects in total.

All other participants met the eligibility criteria and none of exclusion criteria. No protocol deviation was recorded.

Participant‘s age ranged from 23 to 90 years. Median age increased progressively across groups, from 45.04 years in the active TB group to 46.50 years among TB close contacts and 53.75 years within the cohort with autoimmune disease. The active TB group demonstrated the widest distribution, including a single 90-year-old outlier. (Table 1) Overall, 55% of the total was female. Gender distribution varied markedly between groups: the active TB group was predominantly male (73%), all close contacts were female, and the autoimmune cohort showed a near-balanced sex ratio (55% female). All 30 participants in the TB-contact subgroup were female; 23 were occupational contacts and 7 were household contacts. Sex was not an eligibility criterion for study participation, and the all-female distribution reflected the composition of the contact population enrolled during the study period. Because the available database did not include information on eligible contacts who were not enrolled, the reason for the absence of male participants could not be further evaluated (Table 1).

**TABLE 1:** Demographic, residential, and behavioral characteristics of the study population.

Characteristic	Total (*N* = 99)	Active TB (*n* = 49)	Contacts (*n* = 30)	Autoimmune disease (*n* = 20)	*P*-value
Age, median (IQR), years	47.0 (36.0-57.0)	41.0 (34.0-54.0)	47.5 (40.5-55.0)	57.0 (43.5-60.75)	0.013
Sex, *n* (%)					<0.001
Male	45 (45%)	36 (73%)	0 (0%)	9 (45%)	
Female	54 (55%)	13 (27%)	30 (100%)	11 (55%)	
Residence, *n* (%)					0.369
Urban	81 (82%)	38 (78%)	27 (90%)	16 (80%)	
Rural	18 (18%)	11 (22%)	3 (10%)	4 (20%)	
Smoking status, *n* (%)					0.004
Current smoker	40 (40%)	27 (55%)	10 (33%)	3 (15%)	
Former smoker	8 (8%)	5 (10%)	0 (0%)	3 (15%)	
Non-smoker	51 (52%)	17 (35%)	20 (67%)	14 (70%)	

*P*-values were calculated using the Kruskal–Wallis test for age and chi-square/Fisher’s exact test for categorical variables.

### 4.2 Clinical characteristics and immunosuppressive treatment in the autoimmune disease subgroup

The autoimmune disease subgroup included 20 participants, of whom 11/20 (55.0%) were female. The most frequent diagnoses were psoriasis (8/20, 40.0%), ankylosing spondylitis (6/20, 30.0%), Crohn disease (4/20, 20.0%), and rheumatoid arthritis/polyarthritis (2/20, 10.0%). Immunosuppressive treatment consisted predominantly of monoclonal antibody/biologic therapy (17/20, 85.0%), followed by systemic corticosteroids/corticotherapy (2/20, 10.0%) and cytostatic/conventional immunosuppressive therapy (1/20, 5.0%). Specific drug names, corticosteroid doses, biologic class, and disease activity status were not available in the current dataset (Table 2).

**TABLE 2:** Clinical characteristics of the autoimmune disease subgroup.

Characteristics of the Autoimmune disease subgroup (*n* = 20)	
Sex	
Female	11 (55.0%)
Male	9 (45.0%)
Autoimmune diagnosis, based on free-text disease field	
Psoriasis	8 (40.0%)
Ankylosing spondylitis	6 (30.0%)
Crohn disease	4 (20.0%)
Rheumatoid arthritis/polyarthritis	2 (10.0%)
Immunosuppressive treatment recorded at testing	
Monoclonal antibodies / biologic therapy	17 (85.0%)
Systemic corticosteroids / corticotherapy	2 (10.0%)
Cytostatic / conventional immunosuppressive therapy	1 (5.0%)

Percentages were calculated using *n* = 20 as the denominator. Diagnoses were standardized from the free-text disease field. Treatment categories reflect the single treatment code recorded in the uploaded Excel file. Specific drug names, corticosteroid doses, biologic class, and disease activity were not available.

### 4.3 Residential and behavioral characteristics

Residential status was predominantly urban, with 82% of participants residing in urban area. The distribution was similar across study groups, ranging from 78% (in active TB group) to 90% (in TB contacts), indicating no major imbalance in recruitment by residential setting (Table 1).

Regarding smoking status, 52% of participants were non-smokers, 40% were current smokers and 8% were former smokers. The highest proportion of current smokers was observed in individuals with active TB – 55% (Table 1).

### 4.4 Positivity and agreement patterns of IP-10-based assays compared with QFT-plus

**FIGURE 1:**
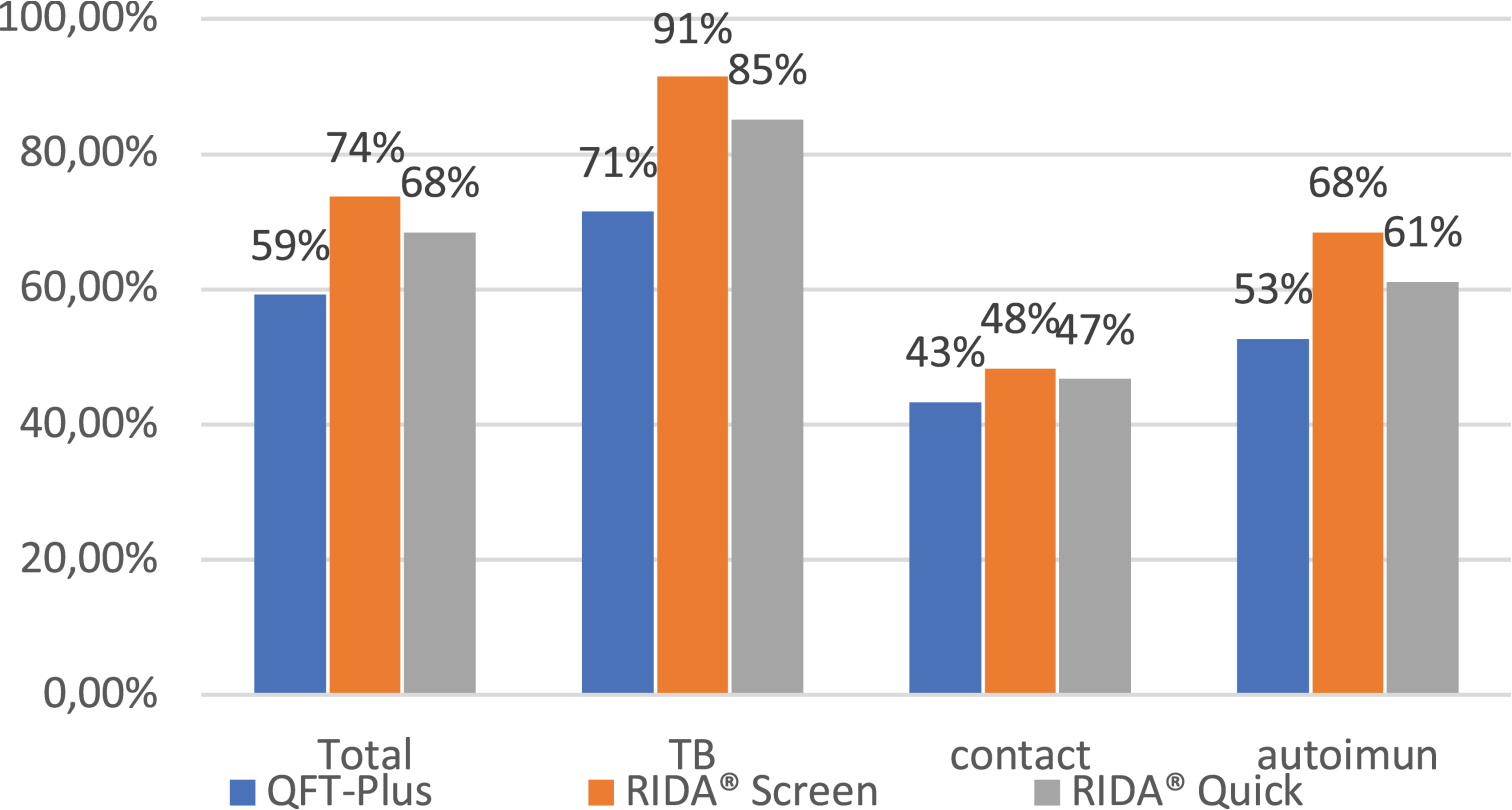
Positivity rate by study group and test type.

In our cohort, both RIDA^®^SCREEN TB and RIDA^®^QUICK TB assays demonstrated higher positivity for Mtb-specific immune response than QFT-Plus, particularly among individuals with culture-confirmed active TB disease. The performance of the two IP-10-based assays was largely comparable, with both showing a consistent trend toward a higher proportion of positive results relative to the QFT-Plus assay (Figure 1). This pattern was observed across positivity and agreement analyses, suggesting that IP-10-mediated assays may capture antigen-specific immune responses that are not consistently detected by IFN-γ-based readouts. Because QFT-Plus is an immunological comparator rather than a definitive infection standard, these findings should be interpreted as differences in immune-response detection and assay concordance rather than proof of superior diagnostic accuracy.

#### 4.4.1 Overall test positivity and microbiological reference comparison

A total of 100 participants were enrolled in the study. Complete paired results for RIDA^®^QUICK TB, RIDA^®^SCREEN TB, and QuantiFERON-TB Gold Plus (QFT-Plus) were available for 99 individuals and were included in the comparative analyses. Detailed agreement analyses between RIDA^®^ QUICK TB and QFT-Plus are provided in Supplementary Appendix A.

Overall test positivity across all study groups is presented in Table 3. The table summarizes the number of positive results obtained with each assay among participants with active TB, TB contacts, and individuals with autoimmune disease, as well as in the total cohort with complete paired results (*N* = 99). Across all groups, both IP-10–based assays yielded a higher number of positive results than QFT-Plus, with the largest differences observed in the active TB subgroup. Because active TB cases were culture-confirmed, the proportion of positive immune-test results in this subgroup was interpreted as sensitivity for active TB-associated immune response. This should not be extrapolated to LTBI sensitivity, as LTBI lacks a microbiological gold standard and was assessed only through comparator-based agreement analyses.

**TABLE 3:** Assay positivity across study groups and case positivity among participants with culture-confirmed active TB.

Study group	*N*	RIDA^®^QUICK TB positive, *n*/*N* (%)	RIDA^®^SCREEN TB positive, *n*/*N* (%)	QFT-Plus positive, *n*/*N* (%)
Active TB	49	42/49 (85.7%)	45/49 (91.8%)	35/49 (71.4%)
TB contacts	30	14/30 (46.7%)	14/30 (46.7%)	13/30 (43.3%)
Autoimmune disease	20	11/20 (55.0%)	13/20 (65.0%)	10/20 (50.0%)
Total	99	67/99 (67.7%)	72/99 (72.7%)	58/99 (58.6%)

In the culture-confirmed active TB subgroup, the proportion of positive immune-test results among culture-positive participants was interpreted as case positivity among participants with culture-confirmed active TB. This estimate is not equivalent to sensitivity for latent tuberculosis infection. Specificity and NPV could not be estimated because no culture-negative reference group was included.

In the full cohort, RIDA^®^QUICK TB showed good overall agreement with QFT-Plus (κ = 0.677), although the McNemar test (*p* = 0.035) demonstrated a significant directional disagreement, with RIDA^®^QUICK TB classifying more samples as positive (12 vs. 3).

In discordant pairs, RIDA^®^QUICK TB produced a positive result more frequently than QFT-Plus, suggesting detection of immune activation not captured by IFN-γ–based readouts. The assay showed high PPA (∼95%), NPV = 0.906, and PPV = 0.821, while NPV was moderate (70.7%), consistent with an expected shift toward increased positivity in an IP-10–driven assay.

##### 4.4.1.1 Subgroup of subjects with active TB disease

In individuals with active TB, agreement between RIDA^®^QUICK TB and QFT-Plus was moderate (κ = 0.471), with an overall accuracy of 81.6%. PPA remained very high (97.1%) and PPV substantial (0.810). NPA, however, was low (0.429), driven by the higher number of RIDA^®^QUICK TB positive/QFT-Plus negative results (8 vs. 1). The McNemar test (*p* = 0.039) confirmed a statistically significant directional imbalance. Importantly, because QFT-Plus is not a definitive reference standard for active TB, these discordant results may reflect antigen-specific immune responses not consistently captured by IFN-γ-based readouts.

##### 4.4.1.2 Subgroup of subjects with TB contact

Among close contacts of TB cases, agreement between the assays was almost perfect (κ = 0.933), with an accuracy of 96.7%. PPA and NPV were 1.0 each, while NPA (0.941) and PPV (0.929) were similarly high. No directional disagreement was observed (McNemar *p* = 1.00). These results reflect excellent assay concordance in a lower-risk population, although interpretation should consider the modest sample size.

##### 4.4.1.3 Subgroup of subjects with autoimmune disease

In participants with autoimmune disease, agreement was moderate (κ = 0.500), with an overall accuracy of 75%. PPA (0.800) and NPV (0.778) were acceptable, while NPA (0.700) and PPV (0.727) were lower than in the other subgroups. No directional asymmetry was identified (McNemar *p* = 1.00). The more variable performance in this subgroup likely reflects underlying immune dysregulation, which can differentially affect IFN-γ release and IP-10 response.

### 4.5 Comparator-based ROC analysis of immunological concordance with QFT-plus

Comparator-based ROC analyses were performed to assess how well the IP-10 assay measurements distinguished QFT-Plus-positive from QFT-Plus-negative immune-response classifications. These curves evaluate concordance with QFT-Plus-defined immunological status and should not be interpreted as diagnostic ROC analyses against confirmed *Mycobacterium tuberculosis* infection.

In the overall cohort, RIDA^®^QUICK TB showed good immunological concordance with QFT-Plus-defined status, with an AUC of 0.828 (95% CI: 0.736–0.919). Concordance varied across clinical subgroups. It was highest among close TB contacts (AUC = 0.971), consistent with the high categorical agreement observed in this group, and lower among participants with active TB (AUC = 0.700) and autoimmune disease (AUC = 0.750). The wider confidence intervals in the autoimmune-disease subgroup indicate limited precision, reflecting the small sample size and biological heterogeneity associated with immune dysregulation.

These AUC values quantify comparator-based immunological concordance between two host-response assays and do not establish diagnostic accuracy against true infection or disease status. Because QFT-Plus is not an independent microbiological reference standard, the ROC-derived estimates should be interpreted only as agreement with QFT-Plus-defined immune status.

#### 4.5.1 Comparison of RIDA^®^QUICK TB with microbiological reference standard in the group of active TB disease subjects

All 49 participants included in the active-TB analysis had culture-confirmed pulmonary tuberculosis. RIDA^®^QUICK TB was positive in 42/49 cases, corresponding to a case positivity estimate of 85.7% (95% CI: approximately 72.8–94.1%). Seven culture-confirmed cases had a negative RIDA^®^QUICK TB result. Detailed results are provided in Supplementary Appendix B.

Because the active-TB subgroup contained no culture-negative reference observations, specificity, predictive values, likelihood ratios, diagnostic accuracy, and ROC/AUC against culture could not be estimated. A culture-referenced ROC curve was therefore not statistically identifiable. The reported proportion should be interpreted only as immune-test positivity among patients with microbiologically confirmed active pulmonary TB and not as diagnostic accuracy for tuberculosis infection or latent tuberculosis infection (Table 3).

#### 4.5.2 Agreement between RIDA^®^SCREEN TB and QFT-plus

RIDA^®^SCREEN TB showed good agreement with QFT-Plus in the overall cohort, with a Cohen’s κ of 0.562. The McNemar test was statistically significant (*p* = 0.0036), indicating a directional imbalance driven by more RIDA^®^SCREEN TB-positive/QFT-Plus-negative discordant classifications than the reverse pattern. Overall, RIDA^®^SCREEN TB demonstrated high positive percent agreement with QFT-Plus and moderate negative percent agreement, reflecting a tendency of the IP-10-based assay to classify more samples as positive than the IFN-γ-based comparator. Comparator-based predictive values were PPV = 0.764 and NPV = 0.889; however, these values reflect agreement with QFT-Plus-defined immune status and should not be interpreted as diagnostic predictive values against confirmed infection or disease. Detailed results are provided in Supplementary Appendix C.

In the active TB subgroup, agreement between RIDA^®^SCREEN TB and QFT-Plus was moderate (κ = 0.365; overall agreement approximately 80%). RIDA^®^SCREEN TB showed high positive percent agreement with QFT-Plus, while negative percent agreement was lower, reflecting more frequent RIDA^®^SCREEN TB-positive/QFT-Plus-negative discordant classifications. The McNemar test (*p* = 0.002) indicated significant directional disagreement. Because QFT-Plus is not a definitive diagnostic reference standard for active TB, these discordant results should be interpreted as differences in immune-response detection between IP-10- and IFN-γ-based platforms, rather than as false-positive or false-negative classifications.

Among close TB contacts, agreement between RIDA^®^SCREEN TB and QFT-Plus was very good (κ = 0.798; overall agreement 90%). Positive percent agreement was 0.923 and negative percent agreement was 0.882, with comparator-based PPV and NPV values of 0.857 and 0.938, respectively. No directional asymmetry was observed using the McNemar test (*p* = 1.00), supporting strong concordance between the two immunological platforms in this subgroup.

In participants with autoimmune disease, agreement was lower and more variable (κ = 0.300; overall agreement 65%). Positive percent agreement was 0.800 and negative percent agreement was 0.500, with comparator-based PPV and NPV values of 0.615 and 0.714, respectively. No statistically significant directional disagreement was detected (McNemar *p* = 0.453). The lower concordance observed in this subgroup may reflect the combined influence of underlying immune dysregulation and immunosuppressive treatment on IFN-γ and IP-10 immune responses.

Across all subgroups, interpretation should remain cautious because of the limited sample size and the comparator-based nature of the analysis. RIDA^®^SCREEN TB showed a tendency toward higher positivity than QFT-Plus, particularly in the active TB subgroup; however, this should be interpreted as immunological discordance between platforms rather than diagnostic superiority. As with RIDA^®^QUICK TB, QFT-Plus-based predictive values reflect the distribution of QFT-Plus positivity in the study population and should not be interpreted as diagnostic performance against confirmed infection.

In the overall cohort, comparator-based ROC analysis showed good immunological concordance between RIDA^®^SCREEN TB and QFT-Plus-defined immune status, with an AUC of 0.767 (95% CI: 0.664–0.869; *p* < 0.001).

Concordance was highest among TB contacts (AUC = 0.903), while comparator-based concordance across the other subgroups was more heterogeneous, consistent with the RIDA^®^QUICK TB/QFT-Plus comparison. These ROC-derived values should be interpreted as measures of immunological concordance with QFT-Plus-defined status rather than as estimates of diagnostic accuracy against a definitive infection standard.

#### 4.5.3 Comparison of RIDA^®^SCREEN TB with microbiological reference standard in the group of active TB disease subjects

RIDA^®^SCREEN TB was positive in 45/49 culture-confirmed active-TB cases, corresponding to a case positivity estimate of 91.8% (95% CI: approximately 80.4%–97.7%). Four culture-confirmed cases had a negative RIDA^®^SCREEN TB result. Detailed results are provided in Supplementary Appendix D.

As all participants in this subgroup were culture-positive, specificity, predictive values, likelihood ratios, overall diagnostic accuracy, and ROC/AUC against culture could not be estimated. This result therefore reflects immune-test positivity in a microbiologically confirmed disease-positive population rather than complete diagnostic performance (Table 3).

#### 4.5.4 Comparison of QFT-plus with microbiological reference standard in the group of active TB disease subjects

QFT-Plus was positive in 35/49 culture-confirmed active-TB cases, corresponding to a case positivity estimate of 71.4% (95% CI: approximately 56.7%–83.4%). Fourteen culture-confirmed cases had a negative QFT-Plus result. Because no culture-negative participants were included, specificity, predictive values, diagnostic accuracy, and ROC/AUC against culture could not be estimated. Detailed results are provided in Supplementary Appendix E.

## 5 Discussions

Across the study cohort, both IP-10-based assays demonstrated higher positivity rates than QFT-Plus, including high case positivity among participants with culture-confirmed active pulmonary TB. Importantly, comparisons with QFT-Plus should be interpreted as immunological concordance rather than definitive diagnostic accuracy, because QFT-Plus and IP-10-based assays measure host immune response and do not directly confirm or exclude viable Mycobacterium tuberculosis. This pattern reflects the biological nature of the assays: IP-10 and IFN-γ release measurements capture antigen-specific immune activation rather than direct detection of viable organisms. Therefore, the active TB subgroup should be interpreted as a microbiologically confirmed positive-control population for exploratory sensitivity estimation, not as evidence that these assays diagnose active TB disease or define sensitivity for LTBI. The biological rationale for evaluating IP-10 as an alternative or complementary readout to IFN-γ is based on both signal amplification and cellular source. Following recognition of *Mycobacterium tuberculosis* antigens, antigen-specific T cells release IFN-γ, which stimulates monocytes, macrophages, dendritic cells, and other responsive cells to produce the chemokine IP-10/CXCL10. Consequently, antigen-induced IP-10 concentrations may be considerably higher than IFN-γ concentrations, providing a broader analytical range and potentially facilitating detection using ELISA, lateral-flow, dried-plasma, or molecular platforms. This amplified downstream signal may be particularly advantageous when IFN-γ responses are low or close to the assay threshold. However, because IP-10 is downstream of several inflammatory pathways and is not specific to tuberculosis, its higher concentration should not be equated automatically with higher clinical specificity or superior diagnostic accuracy. Previous investigations have generally supported IP-10 as a marker of Mycobacterium tuberculosis-specific immune sensitization, although results have varied according to population, assay format, antigen stimulation protocol, and reference definition. Studies in household contacts and contact investigations have reported substantial concordance between antigen-induced IP-10 and established IGRAs, while also identifying discordant responses and possible additional detection near conventional IFN-γ thresholds. Pediatric studies have suggested that IP-10 may provide a technically robust signal in children, including populations in whom blood volume and reduced IFN-γ responses can complicate conventional IGRA interpretation. Other studies have evaluated IP-10 in active TB, HIV infection, and treatment monitoring, but these findings remain heterogeneous and do not establish that IP-10 can distinguish latent infection from active disease. The higher positivity observed with the IP-10 assays in our active-TB subgroup may therefore have several explanations. It could reflect the greater dynamic range of the downstream IP-10 response, differences in assay cut-offs, differences in the cellular pathways captured by the two biomarkers, or reduced IFN-γ production in some patients with active disease. It may also reflect lower analytical specificity or platform-dependent discordance. Because the present study lacks a culture-negative control population and a definitive reference standard for latent infection, these mechanisms cannot be separated. The additional IP-10-positive/QFT-Plus-negative results should therefore be interpreted as discordant immune-response classifications rather than as confirmed additional infections (17, 18).

Importantly, higher positivity rates observed with IP-10–based assays compared with QFT-Plus should not be interpreted as false positivity but rather as detection of antigen-specific immune activation not consistently captured by IFN-γ–only platforms. Consequently, predictive values depend on the underlying prevalence of immunological sensitization and should not be interpreted as direct proxies for true infection. For this reason, comparison with microbiological standard (Mtb culture) is essential to determine whether additional RIDA^®^ positive results represent early immunological responses, low-burden infection, or assay discordance.

In the bacteriologically confirmed active TB subgroup (*N* = 49; all sputum cultures positive), RIDA^®^QUICK TB was positive in 42 of 49 cases, corresponding to a case positivity estimate of 85.7%. Because no culture-negative participants were included, specificity, negative predictive value, positive predictive value, likelihood ratios, overall diagnostic accuracy, and AUC against culture could not be meaningfully estimated.

RIDA^®^SCREEN TB was positive in 45 of 49 culture-confirmed active-TB cases, corresponding to a case positivity estimate of 91.8%. Because the subgroup contained no culture-negative reference observations, specificity, negative predictive value, positive predictive value, likelihood ratios, overall diagnostic accuracy, and AUC against culture could not be meaningfully estimated. Such discrepancies may arise in early infection, low-burden disease, heterogeneous sputum sampling, or temporal differences between immune activation and bacteriological detectability.

The findings of this study are consistent with emerging external evidence supporting the potential value of IP-10 as an alternative or complementary immunological readout for Mycobacterium tuberculosis-specific immune sensitization. A multicenter evaluation by Köffer et al. (19) reported higher sensitivity for both RIDA^®^SCREEN TB and RIDA^®^QUICK TB compared with QFT-Plus, along with strong PPV performance against a composite clinical reference closely matching the performance patterns observed in our cohort. Likewise, recent reviews, including the analysis by Alonzi et al. (20), have identified RIDA^®^ QUICK TB as part of a new generation of IP-10–based IGRAs with improved sensitivity relative to IFN-γ–only platforms. The higher case positivity of both IP-10 assays observed among culture-confirmed active-TB participants in our study aligns with these findings and supports the growing evidence that IP-10 may provide a more robust immunological signal in active and incipient TB. Taken together, these findings indicate that the disease-enriched composition of the active-TB subgroup permits estimation only of the proportion of culture-confirmed cases with positive immune-test results. It does not permit reliable estimation of specificity, predictive values, likelihood ratios, or culture-referenced AUC because no culture-negative reference observations were included. A more complete assessment of diagnostic performance particularly for specificity will require validation in more balanced populations such as close contacts, immunocompromised individuals, and low-risk clinical groups, as well as the application of composite clinical reference standards across the full cohort. These findings underscore the potential of IP-10 assays as valuable complementary tools within TB diagnostic pathways and support further investigation into their utility in diverse epidemiological and immunological contexts. These findings contribute to the growing body of evidence supporting IP-10 as a promising next-generation biomarker in TB immunodiagnostics. Interpretation in the autoimmune-disease subgroup requires particular caution. Systemic corticosteroids, conventional immunosuppressants, anti-TNF agents, and other biologic therapies may reduce antigen-specific T-cell activation, alter IFN-γ production, impair downstream chemokine release, or modify the background inflammatory signal. IP-10 may retain measurable responses in some immunocompromised individuals because of its amplified downstream production, but it is not immune to suppression. Underlying inflammatory disease may also increase unstimulated IP-10 concentrations, potentially affecting the antigen-minus-nil signal and assay threshold. Therefore, the lower and more variable concordance observed in our autoimmune subgroup may reflect both biological heterogeneity and treatment effects.

The current dataset recorded broad treatment categories but not specific agents, corticosteroid doses, treatment duration, disease activity, lymphocyte counts, or timing relative to biologic administration. We were therefore unable to determine whether discordance was associated with a particular immunosuppressive mechanism. Future studies should stratify participants by anti-TNF therapy, non-TNF biologics, Janus kinase inhibitors, corticosteroid exposure, conventional DMARD use, disease activity, and lymphocyte count (21).

## 6 Limitations

This study has several limitations. The subgroup with active TB was disease-enriched, preventing reliable estimation of specificity and NPV. The subgroup with autoimmune diseases was small and various degrees of immune suppression may have influenced test performance. All participants were adults and most of them from urban area of Bucharest, limiting generalizability to rural, pediatric or HIV-infected populations. ROC analyses were performed against an immunological comparator rather than a definitive diagnostic gold standard, limiting inference regarding specificity-driven diagnostic accuracy. The QFT-Plus-based ROC analyses represent comparator-dependent assessments of immunological concordance rather than diagnostic accuracy. QFT-Plus is an indirect host-response assay and cannot establish true infection status. Furthermore, a microbiological ROC analysis could not be performed in the active-TB subgroup because all 49 analyzed participants were culture-positive and no culture-negative reference cases were available. Consequently, only the proportion of positive immune-test results among culture-confirmed active-TB cases could be estimated, whereas specificity, predictive values, likelihood ratios, and culture-referenced AUC were not estimable. Also, the absence of a TB-unexposed control group represents a key limitation, restricting the ability to estimate specificity and negative predictive value and limiting extrapolation to screening settings. Another important limitation inherent to the disease-enriched study design was the absence of a dedicated LTBI-negative control cohort. This reflects a broader methodological challenge in LTBI research, as there is currently no universally accepted gold-standard test capable of definitively excluding latent *Mycobacterium tuberculosis* infection. Consequently, presumed negative controls may still harbor undetected latent infection, introducing potential misclassification bias. Therefore, assay performance was evaluated primarily through comparison with clinically characterized participant groups and the QuantiFERON-TB Gold Plus assay, an immunological test recommended by the WHO for LTBI evaluation. Importantly, QFT-Plus evaluates antigen-specific host immune response rather than the direct presence or absence of *Mycobacterium tuberculosis* bacilli; therefore, a negative QFT-Plus result cannot definitively exclude the presence of *Mycobacterium tuberculosis* bacilli. Accordingly, QFT-Plus was used as a comparator immunological reference rather than a definitive microbiological gold standard. The TB-contact subgroup consisted entirely of women, including 23 occupational contacts and 7 household contacts. Although sex was not an eligibility criterion, the absence of male contacts limits the generalizability of the contact-group findings and prevented evaluation of potential sex-related differences in assay concordance. Information on the sex distribution of eligible contacts who were screened but not enrolled was not available; therefore, the source of this recruitment imbalance could not be determined. Although the initial study objectives included exploration of demographic and clinical factors associated with assay results in the active-TB subgroup, the small number of negative IP-10 assay results did not permit a reliable predictor analysis. Finally, longitudinal follow-up was not performed, preventing evaluation of PPV for progression.

## 7 Clinical implications

The findings of this study suggest that IP-10-based assays may provide useful complementary immunological information in the evaluation of *Mycobacterium tuberculosis* infection. Both RIDA^®^QUICK TB and RIDA^®^SCREEN TB showed high positivity among individuals with culture-confirmed active pulmonary TB and clinically relevant agreement with QFT-Plus across the study population. However, these results should be interpreted as evidence of immune-response detection and assay concordance, rather than as proof of standalone diagnostic accuracy.

In clinical practice, IP-10-based assays may be particularly useful as complementary tools in settings where IFN-γ-based IGRA results are negative, borderline, indeterminate, or difficult to interpret in relation to clinical risk. Their potential value may be relevant in populations with altered immune responses, such as individuals with autoimmune disease or patients receiving immunosuppressive therapy, although this requires further validation in larger and better-characterized cohorts.

The case positivity estimates observed in the culture-confirmed active-TB subgroup reflect positivity among microbiologically confirmed TB cases and should not be extrapolated to latent tuberculosis infection. Similarly, comparisons with QFT-Plus should be interpreted as immunological agreement rather than direct confirmation or exclusion of infection. Therefore, IP-10-based assay results should be integrated with epidemiological exposure, clinical assessment, radiological findings, microbiological testing, and treatment history.

Future multicenter studies including LTBI-focused cohorts, immunocompromised populations, TB-unexposed controls, and longitudinal follow-up are needed to determine specificity, predictive value for progression, and the optimal role of IP-10-based assays in tuberculosis infection screening and preventive treatment decision-making.

## 8 Conclusion

In this comparative evaluation, the IP-10-based RIDA^®^QUICK TB and RIDA^®^SCREEN TB assays demonstrated high positivity among individuals with culture-confirmed active pulmonary TB and clinically relevant agreement with QFT-Plus across the study population. These findings support the potential value of IP-10-mediated immune-response detection as a complementary approach to IFN-γ-based IGRA testing.

The case positivity estimates reported in the culture-confirmed active TB subgroup reflect positivity among microbiologically confirmed TB cases and should not be extrapolated to latent tuberculosis infection, for which no definitive microbiological reference standard exists. Similarly, comparisons with QFT-Plus should be interpreted as immunological agreement and concordance rather than definitive diagnostic accuracy, because QFT-Plus and IP-10-based assays measure host immune response rather than direct detection of viable *Mycobacterium tuberculosis*.

Specificity and negative predictive value could not be estimated in the active-TB subgroup because no culture-negative reference observations were included. This reflects the disease-enriched study design rather than an intrinsic limitation of the assays. When interpreted alongside clinical, radiological, and microbiological data, IP-10-based assays may provide additional immunological information to support tuberculosis infection evaluation.

Overall, these findings align with growing evidence supporting IP-10 as a promising biomarker in TB immunodiagnostics. Further multicenter studies in more diverse and representative populations, including LTBI-focused cohorts with longitudinal follow-up, are needed to define specificity, predictive value, and operational applicability. Until such data are available, RIDA^®^QUICK TB and RIDA^®^SCREEN TB should be regarded as complementary immune-based tools rather than standalone diagnostic tests.

## Data Availability

The data supporting the findings of this study are available from the corresponding author upon reasonable request.
