# Supplementary material for: Comparative performance of IP-10-Based assays for immune detection of Mycobacterium tuberculosis infection: a cross-sectional evaluation
Source: Front Med (Lausanne). 2026 Aug 17;13:1886171. doi: 10.3389/fmed.2026.1886171 (PMC13526965; doi:10.3389/fmed.2026.1886171)
Supplement: Supplementary file 1 [file Supplementary_file_1.docx]

**Appendix A. Statistical Analysis**

Appendix A.1. Agreement Analysis of RIDA®QUICK TB and QFT-Plus Stratified by Clinical Category (Appendix A)

Appendix A.1.1. Crosstabs

| **Case Processing Summary** | | | | | | |
| --- | --- | --- | --- | --- | --- | --- |
|  | Cases | | | | | |
|  | Valid | | Missing | | Total | |
|  | N | Percent | N | Percent | N | Percent |
| RIDA® Quick * QTF-Plus | 99 | 100,0% | 0 | 0,0% | 99 | 100,0% |

| **RIDA® Quick * QTF-Plus Crosstabulation** | | | | | |
| --- | --- | --- | --- | --- | --- |
|  | | | QTF-Plus | | Total |
|  |  |  | 0 | 1 |  |
| RIDA® Quick | 0 | Count | 29 | 3 | 32 |
|  |  | % within RIDA® Quick | 90,6% | 9,4% | 100,0% |
|  |  | % within QTF-Plus | 70,7% | 5,2% | 32,3% |
|  | 1 | Count | 12 | 55 | 67 |
|  |  | % within RIDA® Quick | 17,9% | 82,1% | 100,0% |
|  |  | % within QTF-Plus | **ANNEX**  29,3% | 94,8% | 67,7% |
| Total | | Count | 41 | 58 | 99 |
|  |  | % within RIDA® Quick | 41,4% | 58,6% | 100,0% |
|  |  | % within QTF-Plus | 100,0% | 100,0% | 100,0% |

| **Chi-Square Tests** | | |
| --- | --- | --- |
|  | Value | Exact Sig. (2-sided) |
| McNemar Test |  | ,035^a^ |
| N of Valid Cases | 99 |  |
| a. Binomial distribution used. | | |

| **Symmetric Measures** | | | | | |
| --- | --- | --- | --- | --- | --- |
|  | | Value | Asymp. Std. Error^a^ | Approx. T^b^ | Approx. Sig. |
| Measure of Agreement | Kappa | ,677 | ,075 | 6,870 | ,000 |
| N of Valid Cases | | 99 |  |  |  |
| a. Not assuming the null hypothesis. | | | | | |
| b. Using the asymptotic standard error assuming the null hypothesis. | | | | | |

Appendix A.1.2. Crosstabs

| **Case Processing Summary** | | | | | | | |
| --- | --- | --- | --- | --- | --- | --- | --- |
| G=grup: T:TB activ, C:contact. A: autoimun | | Cases | | | | | |
|  |  | Valid | | Missing | | Total | |
|  |  | N | Percent | N | Percent | N | Percent |
| autoimmune | RIDA® Quick * QTF-Plus | 20 | 100,0% | 0 | 0,0% | 20 | 100,0% |
| contact | RIDA® Quick * QTF-Plus | 30 | 100,0% | 0 | 0,0% | 30 | 100,0% |
| Active TB | RIDA® Quick * QTF-Plus | 49 | 100,0% | 0 | 0,0% | 49 | 100,0% |

| **RIDA® Quick * QTF-Plus Crosstabulation** | | | | | | |
| --- | --- | --- | --- | --- | --- | --- |
| G=grup: T:TB activ, C:contact. A: autoimun | | | | QTF-Plus | | Total |
|  |  |  |  | 0 | 1 |  |
| autoimmune | RIDA® Quick | 0 | Count | 7 | 2 | 9 |
|  |  |  | % within RIDA® Quick | 77,8% | 22,2% | 100,0% |
|  |  |  | % within QTF-Plus | 70,0% | 20,0% | 45,0% |
|  |  | 1 | Count | 3 | 8 | 11 |
|  |  |  | % within RIDA® Quick | 27,3% | 72,7% | 100,0% |
|  |  |  | % within QTF-Plus | 30,0% | 80,0% | 55,0% |
|  | Total | | Count | 10 | 10 | 20 |
|  |  |  | % within RIDA® Quick | 50,0% | 50,0% | 100,0% |
|  |  |  | % within QTF-Plus | 100,0% | 100,0% | 100,0% |
| contact | RIDA® Quick | 0 | Count | 16 | 0 | 16 |
|  |  |  | % within RIDA® Quick | 100,0% | 0,0% | 100,0% |
|  |  |  | % within QTF-Plus | 94,1% | 0,0% | 53,3% |
|  |  | 1 | Count | 1 | 13 | 14 |
|  |  |  | % within RIDA® Quick | 7,1% | 92,9% | 100,0% |
|  |  |  | % within QTF-Plus | 5,9% | 100,0% | 46,7% |
|  | Total | | Count | 17 | 13 | 30 |
|  |  |  | % within RIDA® Quick | 56,7% | 43,3% | 100,0% |
|  |  |  | % within QTF-Plus | 100,0% | 100,0% | 100,0% |
| Active TB | RIDA® Quick | 0 | Count | 6 | 1 | 7 |
|  |  |  | % within RIDA® Quick | 85,7% | 14,3% | 100,0% |
|  |  |  | % within QTF-Plus | 42,9% | 2,9% | 14,3% |
|  |  | 1 | Count | 8 | 34 | 42 |
|  |  |  | % within RIDA® Quick | 19,0% | 81,0% | 100,0% |
|  |  |  | % within QTF-Plus | 57,1% | 97,1% | 85,7% |
|  | Total | | Count | 14 | 35 | 49 |
|  |  |  | % within RIDA® Quick | 28,6% | 71,4% | 100,0% |
|  |  |  | % within QTF-Plus | 100,0% | 100,0% | 100,0% |

| **Chi-Square Tests** | | | |
| --- | --- | --- | --- |
| G=grup: T:TB activ, C:contact. A: autoimun | | Value | Exact Sig. (2-sided) |
| autoimmune | McNemar Test |  | 1,000^a^ |
|  | N of Valid Cases | 20 |  |
| contact | McNemar Test |  | 1,000^a^ |
|  | N of Valid Cases | 30 |  |
| Active TB | McNemar Test |  | ,039^a^ |
|  | N of Valid Cases | 49 |  |
| a. Binomial distribution used. | | | |

| **Symmetric Measures** | | | | | | |
| --- | --- | --- | --- | --- | --- | --- |
| G=grup: T:TB activ, C:contact. A: autoimun | | | Value | Asymp. Std. Error^a^ | Approx. T^b^ | Approx. Sig. |
| autoimmune | Measure of Agreement | Kappa | ,500 | ,193 | 2,247 | ,025 |
|  | N of Valid Cases | | 20 |  |  |  |
| contact | Measure of Agreement | Kappa | ,933 | ,066 | 5,120 | ,000 |
|  | N of Valid Cases | | 30 |  |  |  |
| Active TB | Measure of Agreement | Kappa | ,471 | ,142 | 3,615 | ,000 |
|  | N of Valid Cases | | 49 |  |  |  |
| a. Not assuming the null hypothesis. | | | | | | |
| b. Using the asymptotic standard error assuming the null hypothesis. | | | | | | |

Appendix A.1.3. ROC Curve

| **Case Processing Summary** | |
| --- | --- |
| QTF-Plus | Valid N (listwise) |
| Positive^a^ | 58 |
| Negative | 41 |
| Larger values of the test result variable(s) indicate stronger evidence for a positive actual state. | |
| a. The positive actual state is 1. | |

| **Area Under the Curve** | | | | |
| --- | --- | --- | --- | --- |
| Test Result Variable(s): RIDA® Quick | | | | |
| Area | Std. Error^a^ | Asymptotic Sig.^b^ | Asymptotic 95% Confidence Interval | |
|  |  |  | Lower Bound | Upper Bound |
| ,828 | ,047 | ,000 | ,736 | ,919 |
| The test result variable(s): RIDA® Quick has at least one tie between the positive actual state group and the negative actual state group. Statistics may be biased. | | | | |
| a. Under the nonparametric assumption | | | | |
| b. Null hypothesis: true area = 0.5 | | | | |

| **Coordinates of the Curve** | | |
| --- | --- | --- |
| Test Result Variable(s): RIDA® Quick | | |
| Positive if Greater Than or Equal To^a^ | Sensitivity | 1 - Specificity |
| -1,00 | 1,000 | 1,000 |
| ,50 | ,948 | ,293 |
| 2,00 | ,000 | ,000 |
| The test result variable(s): RIDA® Quick has at least one tie between the positive actual state group and the negative actual state group. | | |
| a. The smallest cutoff value is the minimum observed test value minus 1, and the largest cutoff value is the maximum observed test value plus 1. All the other cutoff values are the averages of two consecutive ordered observed test values. | | |

Appendix A.1.4. ROC Curve

| **Case Processing Summary^b,c^** | | |
| --- | --- | --- |
| G=grup: T:TB activ, C:contact. A: autoimun | QTF-Plus | Valid N (listwise) |
| autoimmune | Positive^a^ | 10 |
|  | Negative | 10 |
| contact | Positive^a^ | 13 |
|  | Negative | 17 |
| Active TB | Positive^a^ | 35 |
|  | Negative | 14 |
| Larger values of the test result variable(s) indicate stronger evidence for a positive actual state. | | |
| a. The positive actual state is 1. | | |
| b. For split file G=grup: T:TB activ, C:contact. A: autoimun = contact, the test variable(s): RIDA® Quick has at least one tie between the positive actual state group and the negative actual state group. | | |
| c. For split file G=grup: T:TB activ, C:contact. A: autoimun = Active TB, the test variable(s): RIDA® Quick has at least one tie between the positive actual state group and the negative actual state group. | | |

Appendix A.1.5. ROC Curve

| **Area Under the Curve^a,d,e^** | | | | | |
| --- | --- | --- | --- | --- | --- |
| Test Result Variable(s): RIDA® Quick | | | | | |
| G=grup: T:TB activ, C:contact. A: autoimun | Area | Std. Error^b^ | Asymptotic Sig.^c^ | Asymptotic 95% Confidence Interval | |
|  |  |  |  | Lower Bound | Upper Bound |
| autoimmune | ,750 | ,115 | ,059 | ,525 | ,975 |
| contact | ,971 | ,034 | ,000 | ,904 | 1,000 |
| Active TB | ,700 | ,093 | ,030 | ,517 | ,883 |
| a. For split file G=grup: T:TB activ, C:contact. A: autoimun = autoimmune, the test result variable(s): RIDA® Quick has at least one tie between the positive actual state group and the negative actual state group. Statistics may be biased. | | | | | |
| b. Under the nonparametric assumption | | | | | |
| c. Null hypothesis: true area = 0.5 | | | | | |
| d. For split file G=grup: T:TB activ, C:contact. A: autoimun = contact, the test result variable(s): RIDA® Quick has at least one tie between the positive actual state group and the negative actual state group. Statistics may be biased. | | | | | |
| e. For split file G=grup: T:TB activ, C:contact. A: autoimun = Active TB, the test result variable(s): RIDA® Quick has at least one tie between the positive actual state group and the negative actual state group. Statistics may be biased. | | | | | |

| **Coordinates of the Curve^a,c,d^** | | | |
| --- | --- | --- | --- |
| Test Result Variable(s): RIDA® Quick | | | |
| G=grup: T:TB activ, C:contact. A: autoimun | Positive if Greater Than or Equal To^b^ | Sensitivity | 1 - Specificity |
| autoimmune | -1,00 | 1,000 | 1,000 |
|  | ,50 | ,800 | ,300 |
|  | 2,00 | ,000 | ,000 |
| contact | -1,00 | 1,000 | 1,000 |
|  | ,50 | 1,000 | ,059 |
|  | 2,00 | ,000 | ,000 |
| Active TB | -1,00 | 1,000 | 1,000 |
|  | ,50 | ,971 | ,571 |
|  | 2,00 | ,000 | ,000 |
| a. For split file G=grup: T:TB activ, C:contact. A: autoimun = autoimmune, the test variable(s): RIDA® Quick has at least one tie between the positive actual state group and the negative actual state group. | | | |
| b. The smallest cutoff value is the minimum observed test value minus 1, and the largest cutoff value is the maximum observed test value plus 1. All the other cutoff values are the averages of two consecutive ordered observed test values. | | | |
| c. For split file G=grup: T:TB activ, C:contact. A: autoimun = contact, the test variable(s): RIDA® Quick has at least one tie between the positive actual state group and the negative actual state group. | | | |
| d. For split file G=grup: T:TB activ, C:contact. A: autoimun = Active TB, the test variable(s): RIDA® Quick has at least one tie between the positive actual state group and the negative actual state group. | | | |

**Appendix B. RIDA®QUICK TB vs. TB Laboratory Assays**

| **RIDA Quick** | **vs** |  |  |  |  |
| --- | --- | --- | --- | --- | --- |
| **N=49** |  | **GENEXPERT** | |  |  |
|  | **N=49** | **Positive** | **Negative** |  |  |
| **RidaQ** | **Positive** | 38 | 4 | 84% |  |
|  | **Negative** | 7 | 0 |  |  |
|  |  |  |  |  |  |
| **N** | **Sensibilitate** | **Specificitate** | **PPV** | **NPV** | **Acuratete** |
| 49 | 0,844 | 0,000 | 0,905 | 0,000 | 0,776 |
|  |  |  |  |  |  |
|  |  | ***MICROSCOPY*** | |  |  |
|  | **N=49** | **Positive** | **Negative** |  |  |
| **RidaQ** | **Positive** | 35 | 7 | 85% | 0,71 |
|  | **Negative** | 6 | 1 |  |  |
|  |  |  |  |  |  |
| **N** | **Sensibilitate** | **Specificitate** | **PPV** | **NPV** | **Acuratete** |
| 49 | 0,854 | 0,125 | 0,833 | 0,143 | 0,735 |
|  |  |  |  |  |  |
|  |  | **CULTURE** | |  |  |
|  | **N=49** | **Positive** | **Negative** |  |  |
| **RidaQ** | **Positive** | 42 | 0 | 86% | 0,86 |
|  | **Negative** | 7 | 0 |  |  |
|  |  |  |  |  |  |
| **N** | **Sensibilitate** | **Specificitate** | **PPV** | **NPV** | **Acuratete** |
| 49 | 0,857 | #DIV/0! | 1,000 | 0,000 | 0,857 |

**Appendix C. RIDA®SCREEN TB vs QTF-Plus**

*Appendix C.1. Crosstabs*

| **Case Processing Summary** | | | | | | |
| --- | --- | --- | --- | --- | --- | --- |
|  | Cases | | | | | |
|  | Valid | | Missing | | Total | |
|  | N | Percent | N | Percent | N | Percent |
| RIDA® Screen * QTF-Plus | 99 | 100,0% | 0 | 0,0% | 99 | 100,0% |

| **RIDA® Screen * QTF-Plus Crosstabulation** | | | | | |
| --- | --- | --- | --- | --- | --- |
|  | | | QTF-Plus | | Total |
|  |  |  | 0 | 1 |  |
| RIDA® Screen | 0 | Count | 24 | 3 | 27 |
|  |  | % within RIDA® Screen | 88,9% | 11,1% | 100,0% |
|  |  | % within QTF-Plus | 58,5% | 5,2% | 27,3% |
|  | 1 | Count | 17 | 55 | 72 |
|  |  | % within RIDA® Screen | 23,6% | 76,4% | 100,0% |
|  |  | % within QTF-Plus | 41,5% | 94,8% | 72,7% |
| Total | | Count | 41 | 58 | 99 |
|  |  | % within RIDA® Screen | 41,4% | 58,6% | 100,0% |
|  |  | % within QTF-Plus | 100,0% | 100,0% | 100,0% |

| **Chi-Square Tests** | | |
| --- | --- | --- |
|  | Value | Exact Sig. (2-sided) |
| McNemar Test |  | ,003^a^ |
| N of Valid Cases | 99 |  |
| a. Binomial distribution used. | | |

| **Symmetric Measures** | | | | | |
| --- | --- | --- | --- | --- | --- |
|  | | Value | Asymp. Std. Error^a^ | Approx. T^b^ | Approx. Sig. |
| Measure of Agreement | Kappa | ,562 | ,083 | 5,873 | ,000 |
| N of Valid Cases | | 99 |  |  |  |
| a. Not assuming the null hypothesis. | | | | | |
| b. Using the asymptotic standard error assuming the null hypothesis. | | | | | |

*Appendix C.2. Crosstabs*

| **Case Processing Summary** | | | | | | | |
| --- | --- | --- | --- | --- | --- | --- | --- |
| G=grup: T:TB activ, C:contact. A: autoimun | | Cases | | | | | |
|  |  | Valid | | Missing | | Total | |
|  |  | N | Percent | N | Percent | N | Percent |
| autoimmune | RIDA® Screen * QTF-Plus | 20 | 100,0% | 0 | 0,0% | 20 | 100,0% |
| contact | RIDA® Screen * QTF-Plus | 30 | 100,0% | 0 | 0,0% | 30 | 100,0% |
| Active TB | RIDA® Screen * QTF-Plus | 49 | 100,0% | 0 | 0,0% | 49 | 100,0% |

| **RIDA® Screen * QTF-Plus Crosstabulation** | | | | | | |
| --- | --- | --- | --- | --- | --- | --- |
| G=grup: T:TB activ, C:contact. A: autoimun | | | | QTF-Plus | | Total |
|  |  |  |  | 0 | 1 |  |
| autoimmune | RIDA® Screen | 0 | Count | 5 | 2 | 7 |
|  |  |  | % within RIDA® Screen | 71,4% | 28,6% | 100,0% |
|  |  |  | % within QTF-Plus | 50,0% | 20,0% | 35,0% |
|  |  | 1 | Count | 5 | 8 | 13 |
|  |  |  | % within RIDA® Screen | 38,5% | 61,5% | 100,0% |
|  |  |  | % within QTF-Plus | 50,0% | 80,0% | 65,0% |
|  | Total | | Count | 10 | 10 | 20 |
|  |  |  | % within RIDA® Screen | 50,0% | 50,0% | 100,0% |
|  |  |  | % within QTF-Plus | 100,0% | 100,0% | 100,0% |
| contact | RIDA® Screen | 0 | Count | 15 | 1 | 16 |
|  |  |  | % within RIDA® Screen | 93,8% | 6,2% | 100,0% |
|  |  |  | % within QTF-Plus | 88,2% | 7,7% | 53,3% |
|  |  | 1 | Count | 2 | 12 | 14 |
|  |  |  | % within RIDA® Screen | 14,3% | 85,7% | 100,0% |
|  |  |  | % within QTF-Plus | 11,8% | 92,3% | 46,7% |
|  | Total | | Count | 17 | 13 | 30 |
|  |  |  | % within RIDA® Screen | 56,7% | 43,3% | 100,0% |
|  |  |  | % within QTF-Plus | 100,0% | 100,0% | 100,0% |
| Active TB | RIDA® Screen | 0 | Count | 4 | 0 | 4 |
|  |  |  | % within RIDA® Screen | 100,0% | 0,0% | 100,0% |
|  |  |  | % within QTF-Plus | 28,6% | 0,0% | 8,2% |
|  |  | 1 | Count | 10 | 35 | 45 |
|  |  |  | % within RIDA® Screen | 22,2% | 77,8% | 100,0% |
|  |  |  | % within QTF-Plus | 71,4% | 100,0% | 91,8% |
|  | Total | | Count | 14 | 35 | 49 |
|  |  |  | % within RIDA® Screen | 28,6% | 71,4% | 100,0% |
|  |  |  | % within QTF-Plus | 100,0% | 100,0% | 100,0% |

| **Chi-Square Tests** | | | |
| --- | --- | --- | --- |
| G=grup: T:TB activ, C:contact. A: autoimun | | Value | Exact Sig. (2-sided) |
| autoimmune | McNemar Test |  | ,453^a^ |
|  | N of Valid Cases | 20 |  |
| contact | McNemar Test |  | 1,000^a^ |
|  | N of Valid Cases | 30 |  |
| Active TB | McNemar Test |  | ,002^a^ |
|  | N of Valid Cases | 49 |  |
| a. Binomial distribution used. | | | |

| **Symmetric Measures** | | | | | | |
| --- | --- | --- | --- | --- | --- | --- |
| G=grup: T:TB activ, C:contact. A: autoimun | | | Value | Asymp. Std. Error^a^ | Approx. T^b^ | Approx. Sig. |
| autoimmune | Measure of Agreement | Kappa | ,300 | ,203 | 1,406 | ,160 |
|  | N of Valid Cases | | 20 |  |  |  |
| contact | Measure of Agreement | Kappa | ,798 | ,110 | 4,382 | ,000 |
|  | N of Valid Cases | | 30 |  |  |  |
| Active TB | Measure of Agreement | Kappa | ,364 | ,138 | 3,300 | ,001 |
|  | N of Valid Cases | | 49 |  |  |  |
| a. Not assuming the null hypothesis. | | | | | | |
| b. Using the asymptotic standard error assuming the null hypothesis. | | | | | | |

*Appendix C.3. ROC Curve*

| **Case Processing Summary** | |
| --- | --- |
| QTF-Plus | Valid N (listwise) |
| Positive^a^ | 58 |
| Negative | 41 |
| Larger values of the test result variable(s) indicate stronger evidence for a positive actual state. | |
| a. The positive actual state is 1. | |

| **Area Under the Curve** | | | | |
| --- | --- | --- | --- | --- |
| Test Result Variable(s): RIDA® Screen | | | | |
| Area | Std. Error^a^ | Asymptotic Sig.^b^ | Asymptotic 95% Confidence Interval | |
|  |  |  | Lower Bound | Upper Bound |
| ,767 | ,052 | ,000 | ,664 | ,869 |
| The test result variable(s): RIDA® Screen has at least one tie between the positive actual state group and the negative actual state group. Statistics may be biased. | | | | |
| a. Under the nonparametric assumption | | | | |
| b. Null hypothesis: true area = 0.5 | | | | |

| **Coordinates of the Curve** | | |
| --- | --- | --- |
| Test Result Variable(s): RIDA® Screen | | |
| Positive if Greater Than or Equal To^a^ | Sensitivity | 1 - Specificity |
| -1,00 | 1,000 | 1,000 |
| ,50 | ,948 | ,415 |
| 2,00 | ,000 | ,000 |
| The test result variable(s): RIDA® Screen has at least one tie between the positive actual state group and the negative actual state group. | | |
| a. The smallest cutoff value is the minimum observed test value minus 1, and the largest cutoff value is the maximum observed test value plus 1. All the other cutoff values are the averages of two consecutive ordered observed test values. | | |

*Appendix C.4. ROC Curve*

| **Case Processing Summary^b,c^** | | |
| --- | --- | --- |
| G=grup: T:TB activ, C:contact. A: autoimun | QTF-Plus | Valid N (listwise) |
| autoimmune | Positive^a^ | 10 |
|  | Negative | 10 |
| contact | Positive^a^ | 13 |
|  | Negative | 17 |
| Active TB | Positive^a^ | 35 |
|  | Negative | 14 |
| Larger values of the test result variable(s) indicate stronger evidence for a positive actual state. | | |
| a. The positive actual state is 1. | | |
| b. For split file G=grup: T:TB activ, C:contact. A: autoimun = contact, the test variable(s): RIDA® Screen has at least one tie between the positive actual state group and the negative actual state group. | | |
| c. For split file G=grup: T:TB activ, C:contact. A: autoimun = Active TB, the test variable(s): RIDA® Screen has at least one tie between the positive actual state group and the negative actual state group. | | |

*Appendix C.5. ROC Curve*

| **Area Under the Curve^a,d,e^** | | | | | |
| --- | --- | --- | --- | --- | --- |
| Test Result Variable(s): RIDA® Screen | | | | | |
| G=grup: T:TB activ, C:contact. A: autoimun | Area | Std. Error^b^ | Asymptotic Sig.^c^ | Asymptotic 95% Confidence Interval | |
|  |  |  |  | Lower Bound | Upper Bound |
| autoimmune | ,650 | ,126 | ,257 | ,403 | ,897 |
| contact | ,903 | ,063 | ,000 | ,779 | 1,000 |
| Active TB | ,643 | ,096 | ,121 | ,454 | ,832 |
| a. For split file G=grup: T:TB activ, C:contact. A: autoimun = autoimmune, the test result variable(s): RIDA® Screen has at least one tie between the positive actual state group and the negative actual state group. Statistics may be biased. | | | | | |
| b. Under the nonparametric assumption | | | | | |
| c. Null hypothesis: true area = 0.5 | | | | | |
| d. For split file G=grup: T:TB activ, C:contact. A: autoimun = contact, the test result variable(s): RIDA® Screen has at least one tie between the positive actual state group and the negative actual state group. Statistics may be biased. | | | | | |
| e. For split file G=grup: T:TB activ, C:contact. A: autoimun = Active TB, the test result variable(s): RIDA® Screen has at least one tie between the positive actual state group and the negative actual state group. Statistics may be biased. | | | | | |

| **Coordinates of the Curve^a,c,d^** | | | |
| --- | --- | --- | --- |
| Test Result Variable(s): RIDA® Screen | | | |
| G=grup: T:TB activ, C:contact. A: autoimun | Positive if Greater Than or Equal To^b^ | Sensitivity | 1 - Specificity |
| autoimmune | -1,00 | 1,000 | 1,000 |
|  | ,50 | ,800 | ,500 |
|  | 2,00 | ,000 | ,000 |
| contact | -1,00 | 1,000 | 1,000 |
|  | ,50 | ,923 | ,118 |
|  | 2,00 | ,000 | ,000 |
| Active TB | -1,00 | 1,000 | 1,000 |
|  | ,50 | 1,000 | ,714 |
|  | 2,00 | ,000 | ,000 |
| a. For split file G=grup: T:TB activ, C:contact. A: autoimun = autoimmune, the test variable(s): RIDA® Screen has at least one tie between the positive actual state group and the negative actual state group. | | | |
| b. The smallest cutoff value is the minimum observed test value minus 1, and the largest cutoff value is the maximum observed test value plus 1. All the other cutoff values are the averages of two consecutive ordered observed test values. | | | |
| c. For split file G=grup: T:TB activ, C:contact. A: autoimun = contact, the test variable(s): RIDA® Screen has at least one tie between the positive actual state group and the negative actual state group. | | | |
| d. For split file G=grup: T:TB activ, C:contact. A: autoimun = Active TB, the test variable(s): RIDA® Screen has at least one tie between the positive actual state group and the negative actual state group. | | | |

**Appendix D. RIDA®SCREEN TB vs. TB Laboratory Assays**

| **RIDA SCREEN** | **vs** |  |  |  |  |
| --- | --- | --- | --- | --- | --- |
| **N=49** |  | **GENEXPERT** | |  |  |
|  | **N=49** | **Positive** | **Negative** |  |  |
| **RIDASCREEN** | **Positive** | 41 | 4 | 91% |  |
|  | **Negative** | 4 | 0 |  |  |
|  |  |  |  |  |  |
| **N** | **Sensibilitate** | **Specificitate** | **PPV** | **NPV** | **Acuratete** |
| 49 | 0,911 | 0,000 | 0,911 | 0,000 | 0,837 |
|  |  |  |  |  |  |
|  |  |  |  |  |  |
|  |  | ***MICROSCOPY*** | |  |  |
|  | **N=49** | **Positive** | **Negative** |  |  |
| **RIDASCREEN** | **Positive** | 38 | 7 | 93% | 0,76 |
|  | **Negative** | 3 | 1 |  |  |
|  |  |  |  |  |  |
| **N** | **Sensibilitate** | **Specificitate** | **PPV** | **NPV** | **Acuratete** |
| 49 | 0,927 | 0,125 | 0,844 | 0,250 | 0,796 |
|  |  |  |  |  |  |
|  |  |  |  |  |  |
|  |  | **CULTURE** | |  |  |
|  | **N=49** | **Positive** | **Negative** |  |  |
| **RIDASCREEN** | **Positive** | 45 | 0 | 92% | 0,9 |
|  | **Negative** | 4 | 0 |  |  |
|  |  |  |  |  |  |
| **N** | **Sensibilitate** | **Specificitate** | **PPV** | **NPV** | **Acuratete** |
| 49 | 0,918 | - | 1,000 | 0,000 | 0,918 |
|  |  |  |  |  |  |

**Appendix E. QFT-Plus vs. TB Laboratory Assays**

| **QFT-Plus** | **vs** |  |  |  |  |
| --- | --- | --- | --- | --- | --- |
| N=49 |  | **GENEXPERT** | |  |  |
|  | **N=49** | **Positive** | **Negative** |  |  |
| **QFT-Plus** | **Positive** | 33 | 2 | 73% |  |
|  | **Negative** | 12 | 2 |  |  |
|  |  |  |  |  |  |
| **N** | **Sensibilitate** | **Specificitate** | **PPV** | **NPV** | **Acuratete** |
| 49 | 0,733 | 0,500 | 0,943 | 0,143 | 0,714 |
|  |  |  |  |  |  |
|  |  | ***MICROSCOPY*** | |  |  |
|  | **N=49** | **Positive** | **Negative** |  |  |
| **QFT-Plus** | **Positive** | 29 | 6 | 71% | 0,58 |
|  | **Negative** | 12 | 2 |  |  |
|  |  |  |  |  |  |
| **N** | **Sensibilitate** | **Specificitate** | **PPV** | **NPV** | **Acuratete** |
| 49 | 0,707 | 0,250 | 0,829 | 0,143 | 0,633 |
|  |  |  |  |  |  |
|  |  | **CULTURE** | |  |  |
|  | **N=49** | **Positive** | **Negative** |  |  |
| **QFT-Plus** | **Positive** | 35 | 0 | 71% | 0,7 |
|  | **Negative** | 14 | 0 |  |  |
|  |  |  |  |  |  |
| **N** | **Sensibilitate** | **Specificitate** | **PPV** | **NPV** | **Acuratete** |
| 49 | 0,714 | - | 1,000 | 0,000 | 0,714 |
